# The ongoing violence against women: Female Genital Mutilation/Cutting

**DOI:** 10.1186/s12978-016-0159-3

**Published:** 2016-04-18

**Authors:** Jacinta K. Muteshi, Suellen Miller, José M. Belizán

**Affiliations:** Population Council, Nairobi, Kenya; Department Obstetrics, Gynecology, Reproductive Sciences, Bixby Center for Reproductive Health, University of California, San Francisco, USA; Institute for Clinical Effectiveness and Health Policy, Buenos Aires, Argentina

## Abstract

Female Genital Mutilation/Cutting (FGM/C) comprises different practices involving cutting, pricking, removing and sometimes sewing up external female genitalia for non-medical reasons. The practice of FGM/C is highly concentrated in a band of African countries from the Atlantic coast to the Horn of Africa, in areas of the Middle East such as Iraq and Yemen, and in some countries in Asia like Indonesia. Girls exposed to FGM/C are at risk of immediate physical consequences such as severe pain, bleeding, and shock, difficulty in passing urine and faeces, and sepsis. Long-term consequences can include chronic pain and infections. FGM/C is a deeply entrenched social norm, perpetrated by families for a variety of reasons, but the results are harmful. FGM/C is a human rights issue that affects girls and women worldwide. The practice is decreasing, due to intensive advocacy activities of international, national, and grassroots agencies. An adolescent girl today is about a third less likely to be cut than 30 years ago. However, the rates of abandonment are not high enough, and change is not happening as rapidly as necessary. Multiple interventions have been implemented, but the evidence base on what works is lacking. We in reproductive health must work harder to find strategies to help communities and families abandon these harmful practices.

More than 200,000,000 girls and woman have undergone Female Genital Mutilation and Cutting (FGM/C) in 30 high prevalence countries, mainly in Africa, South Asia, and the Middle East. It is estimated that 30 million girls under the age of 15 are at risk of FGM/C over the next decade [1]. National surveys show that prevalence varies widely between and within countries; however, over half of the 200,000,000 girls/women with FGM/C live in Indonesia, Egypt, and Ethiopia. 44 million are girls below age 15. In most of the countries, the majority of girls were cut before age 5; in Yemen, 85 per cent of girls experienced the practice within their first week of life [1].

Available data from large-scale representative surveys show that the practice of FGM/C is highly concentrated in a band of African countries from the Atlantic coast to the Horn of Africa, in areas of the Middle East such as Iraq and Yemen, and in some countries in Asia like Indonesia [2]. However, FGM/C is a human rights issue that affects girls and women worldwide. Evidence suggests that FGM/C exists in some places in South America such as Colombia [3] and elsewhere in the world including in India [4], Malaysia [5], Oman [6], Saudi Arabia [7], and the United Arab Emirates [8], with large variations in terms of the type performed, circumstances surrounding the practice and size of the affected population groups [1]. The practice is also found in pockets of Europe, Australia and North America, which, for the last several decades, have been destinations for migrants from countries where the practice still occurs [1].

By 2050, nearly 1 in 3 births worldwide will occur in the 30 countries in Africa and the Middle East where FGM/C is concentrated, and nearly 500 million more girls and women will be living in these countries than there are today. In Somalia alone, where FGM/C prevalence stands at 98 per cent, the number of girls and women will more than double. In Mali, where prevalence is 89 per cent, the female population will nearly triple [1].

FGM/C is a deeply entrenched social norm. Communities practice FGM/C in the belief that it will ensure a girl’s proper marriage, chastity, beauty or family honour. Some also associate it with religious beliefs, although no religious scriptures require it. The practice is such a powerful social norm that families have their daughters cut even when they are aware of the harm it can cause. If families were to stop practicing on their own they would risk the marriage prospects of their daughter as well as the family’s status [9].

FGM/C comprises different practices involving cutting, pricking, removing and sometimes sewing up external female genitalia for non-medical reasons. WHO has broadly classified the types of procedure performed into four categories; Type 1, clitoridectomy, involves partial or total removal of the clitoris and/or the prepuce. Type 2, excision, involves partial or total removal of the clitoris and the labia minora, with or without excision of the labia majora. Type 3, infibulation, involves narrowing of the vaginal orifice with creation of a covering seal by cutting and appositioning the labia minora and/or the labia majora, with or without excision of the clitoris. Infibulation is considered the most invasive type of FGM/C. Defibulation, opening of the covering seal, is often necessary prior to childbirth. Reinfibulation refers to the recreation of an infibulation after defibulation. Type 4, other, involves all other harmful procedures to the female genitalia for non-medical purposes, for example: pricking, piercing, incising, scraping and cauterizing [2].

For the vast majority of girls a traditional practitioner, usually a woman, performs FGM/C often without any form of anaesthesia or analgesia using non-sterile instruments such as scissors, razor blades or broken glass [10, 11]. While in some places the practice has been medicalized, to reduce health risks, FGM/C is always traumatic, and may be associated with a series of health risks with short- and long-term consequences. Girls exposed to FGM/C are at risk of immediate physical consequences such as severe pain, bleeding, and shock, difficulty in passing urine and faeces, and infections. Long term consequences can include chronic pain and infections [12]. In general, the consequences are similar for FGM/C Type I, II, and III, but they tend to be more severe and more prevalent the more extensive the procedure [12]. A systematic review of the health complications of FGM/C identified a range of obstetrical problems, the most common being prolonged labour and/or obstruction, episiotomies and perineal tears, post partum haemorrhage, and maternal and foetal death [13]. A study investigating 28,393 women attending 28 obstetric centres in several African countries concluded that women with FGM/C are significantly more likely than intact women to have adverse obstetric outcomes such as a caesarean section, postpartum haemorrhage, extended maternal hospital stay, infant resuscitation, stillbirth or early neonatal death, and low birthweight. FGM is estimated to lead to an extra one to two perinatal deaths per 100 deliveries [14]. Consequences are graded according to the type of FGM.

For many girls and women, undergoing FGM/C is a traumatic experience that leaves a lasting psychological mark and may adversely affect their mental health. In fact, several psychological and psychosomatic disorders such as disordered eating and sleeping habits have been attributed to FGM/C. Disordered eating habits include loss of appetite, weight loss or excessive weight gain, and disordered sleeping habits include sleeplessness and recurring nightmares [15]. There are also reports of posttraumatic stress disorder, anxiety, depression, and memory loss associated with FGM/C [12].

FGM/C is recognized as a harmful practice which violates the human rights – civil, cultural, economic, political and social – of girls and women [12]. Further, FGM/C is a stark manifestation of gender inequality and discrimination “related to the historical subjugation and suppression on women” [16]. By extension, it is hypothesized that changing beliefs about women’s rights is a key to its abandonment [12]; with the United Nations General Assembly (2012), the UN Commission on the Status of Women (2010), the African Union and the European Union (2011-2012) and national governments calling for intensified global efforts to support the abandonment/elimination of FGM/C [17, 18]. In September 2015, the Sustainable Development Goals were created, FGM/C elimination was included under Goal 5, to eliminate harmful practices, including child, early and forced marriage and FGM/C [19].

Change is slow, but occurring, and globally rates are decreasing. Overall, an adolescent girl today is about a third less likely to be cut than 30 years ago. Kenya and Tanzania have seen rates drop to a third of their levels three decades ago through a combination of community activism and legislation. In the Central African Republic, Iraq, Liberia and Nigeria, prevalence has dropped by as much as half. Attitudes are also changing: recent data show that the majority of people in the countries where FGM is practiced believe it should end, but continue to compel their daughters to undergo the procedure because of strong social pressure [1].

Countries, communities, and individuals go through transitional stages in terms of desire to adhere to FGM/C, to contemplate abandoning the practice, and to abandon the practice. The readiness to abandon FGM/C varies across and within countries. For example, Somalia is a country with a high prevalence (98 %) and strong desire to adhere to the practice; in Egypt, two-thirds of women want to adhere, but almost one-quarter want to abandon; in Nigeria, almost equal proportions (about 40 %) want to adhere and to abandon respectively, with 14 % “reluctantly adhering”, and 13 % contemplating abandonment [20]. Globally, the rate of decline is inadequate to prevent large numbers of girls from FGM/C.

A 2009 systematic review on effectiveness of interventions designed to reduce the prevalence of FGM/C, identified 3,667 publications on the topic; only six studies fulfilled the inclusion criteria [21]. All studies were controlled before-and-after studies conducted in Africa, Burkina Faso, Egypt, Ethiopia/Kenya, Mali, Nigeria, and Senegal. Collectively, the studies involved 6,803 participants at entry. All studies compared an intervention with no intervention (except one which included an education module). There was great variation in prevalence, ethnicity, religion, and education in the settings. Two of the interventions were directed at the individual level, and four at the community level. The first individually-based study consisted of educational activities delivered to health personnel in Mali, who learned about context and local rationale of FGM/C as well as the different types of FGM/C and its health complications. The other individually-based study took place in Egypt and involved female university students, who received information about reproductive health, including FGM/C. The multifaceted, community-based intervention in Kenya was delivered in a Somali refugee camp, and six village communities in Ethiopia received a nearly identical intervention, consisting of community meetings, theatre performances, video sessions, and mass media activities. In Nigeria, multifaceted community activities, including multimedia and gender equity action plan development, were delivered at three community levels. A Community empowerment intervention took place first in Senegal and subsequentially replicated in Burkina Faso. It consisted of educational sessions in human rights, problem solving, environmental hygiene, and women’s health. The most frequently reported outcomes of the projects were changes in beliefs/attitudes, knowledge/awareness, and intentions concerning FGM/C. Less frequently reported outcomes were self-reported prevalence, behaviours such as talking to others about FGM/C, perceptions regarding spouse’s disapproval of FGM/C, and participants’ regrets of having had their daughters cut. The effect estimates suggest that 1) training health personnel likely produced no effects in knowledge or beliefs/attitudes about FGM/C; 2) educating female students may possibly have led to a small increase in knowledge/awareness about FGM/C; 3) multifaceted community activities may possibly have increased the proportion of participants having favourable knowledge and intentions about FGM/C; 4) community empowerment through education may possibly have positively affected prevalence of FGM/C, participants’ knowledge about the consequences of FGM/C, and regrets about having had their daughters cut. However, the authors stated that low quality of the body of evidence affects the interpretation of results [21, 22].

An impressive range of documented programmatic, research and policy interventions are being implemented to encourage communities, families, and/or individuals to abandon FGM/C, led by a range of national and international Non-governmental organization (NGOs); health, human rights and legal organizations; women’s organizations; UN agencies; and immigrant and refugee service organizations. The main intervention strategies have either been framed as multi-faceted or as standalone activities and have encompassed advocacy/education interventions to community, political and religious leaders, legislative interventions, capacity building interventions, health care interventions, media interventions, and community dialogue.

There remains much to learn from the decades of interventions completed and those currently underway. One important lesson has been that single issue approaches will not eliminate FGM/C, given the diversity of practicing communities; rather community specific, multi-faceted programming that responds to the dynamism of individuals, groups and communities; recognizes the varied patterns of decision-making and the combined influences of education, the economy, politics, law, religion and social environments will be better positioned to inform efforts towards FGM/C abandonment. Lastly, efforts need to be linked to strengthening women’s reproductive and sexual rights.

Historically low levels of funding for FGM/C research has meant that evidence-based knowledge about which combinations and sequences of interventions have had the most impact on behaviour change, through which causal pathways, and which demonstrate the potential for sustainable focused strategies is lacking. Further, given the contextually specific findings in which FHM/C occurs makes generalizations difficult. The lack of theory-based interventions; the existence of data with poor validity because of limited methodological development; and the fragmented documentation of research uptake and use for policy and programming are all obstacles to be overcome [21, 22].

There are multiple grassroots, community, women’s, human rights’, legal, governmental, NGO, and research groups working to stop FGM/C. Emerging work by the Population Council, a New York based International NGO, is seeking to strengthen the evidence base on FGM/C with rigorous research in several areas including:

- Understanding FGM/C drivers, determinants, and trends across a range of contexts.
- Understanding the implementation processes and assessing the effects of types of FGM/C abandonment interventions, their wider impacts on girls’ and women’s lives, and their sustainability
- Improving understanding of the wider impacts of FGM/C and the potential for FGM/C abandonment interventions to impact more broadly on girls, women, their families and communities.
- Improving the measurement of FGM/C status, prevalence, norms and norms changes [23].

The efforts to end FGM/C is global and slowly making progress, but the rates of abandonment are not high enough, and change is not happening as rapidly as necessary. We in the field of Reproductive Health must work harder to find methods to help communities and families abandon this harmful practice, which violates girls’ human rights and often leaves them physically and emotionally traumatized.
